# Analysis of the Profile of Volatile Compounds During the Growing Season in Leaves of *Aesculus* Trees Differing in Susceptibility to Horse Chestnut Leaf Miner (*Cameraria ohridella* Deschka & Dimić)

**DOI:** 10.3390/molecules30030518

**Published:** 2025-01-23

**Authors:** Maja Paterska, Hanna Bandurska, Mariusz Dziadas, Bogna Zawieja, Tamara Chadzinikolau

**Affiliations:** 1 Department of Plant Physiology, Poznan University of Life Sciences, Wołyńska 35, 60-637 Poznan, Poland; tamara.chadzinikolau@up.poznan.pl; 2 Faculty of Chemistry, University of Wroclaw, 14F. Joliot-Curie St., 50-383 Wroclaw, Poland; mariusz.dziadas@uwr.edu.pl; 3 Department of Mathematical and Statistical Methods, Poznan University of Life Sciences, Wojska Polskiego 28, 60-637 Poznan, Poland; bogna.zawieja@up.poznan.pl

**Keywords:** *Aesculus*, *Cameraria ohridella*, VOCs

## Abstract

The invasive pest *Cameraria ohridella* annually colonizes trees of the genus *Aesculus* across Europe, causing dark brown damage called mines that gradually cover the leaf surface. This study aimed to compare the VOC profiles emitted by chestnut tree leaves with varying susceptibility to the pest and assess whether these profiles change due to larval feeding. The research involved a susceptible tree (*Ae. turbinata*) and resistant trees (*Ae. glabra* and *Ae. parviflora*). Over three growing seasons (2014, 2015, 2016), leaf damage and the profile of VOCs were analyzed biweekly from May to September. Leaf damage increased progressively in *Ae. turbinata* during all seasons. The VOC profiles differed both qualitatively and quantitatively among the trees and across years. More VOCs were identified in 2014 and 2015 than in 2016. The susceptible *Ae. turbinata* exhibited the highest VOC diversity in 2014, aligning with severe leaf damage—profiles of VOCs in *Ae. turbinata* were different from resistant trees. Statistical analysis revealed that in 2014 and 2015, differences in the profile of VOCs between susceptible and resistant trees were apparent near pest-feeding dates. In 2016, VOCs significantly distinguished the profile of susceptible trees that were present before the appearance of the first generation of the pest.

## 1. Introduction

The horse chestnut leaf miner (*Cameraria ohridella* Deschka & Dimić) is a moth of the *Gracillariidae* family. This is a leaf-mining insect—the larvae feed on the leaf tissue and make long mines inside the chlorenchyma of the host plant. As a result of feeding, the formation of round dark brown damage called mines gradually occupies more and more leaf area. In extreme cases, it destroys leaves and causes complete tree defoliation [1,2]. The primary host of horse chestnut leaf miner is white chestnut (*Aesculus hippocastanum*). A species very susceptible to pests is the Japanese horse chestnut (*Aesculus turbinata*). According to recent studies, the resistant species are *Aesculus parviflora*, *Aesculus wilsoni*, *Aesculus assamica*, *Aesculus californica*, and *Aesculus indica.* Moreover, it was shown that some species (*Aesculus sylvatica*, *Aesculus pavia*, *Aesculus flava*, and *Aesculus glabra*) are colonized by females that deposit eggs on the leaves’ surface, but most larvae die during their development [3]. The rate of expansion of chestnut leaf miners is astonishing. It was first recorded in Macedonia in 1985, and since then, this moth has spread to most of Europe as well as western and central Asia [4,5,6,7]. The likely reason for the success of this herbivorous insect is the low efficiency of their natural enemies (birds, arthropods, and parasitoids), their high fertility, and the occurrence of several generations during one year, as well as their ability to use wind and passive movement as means of transport [8,9].

High insolation and high temperatures in summer probably stimulate females to lay eggs faster [10]. Furthermore, lower air temperature, fewer sun hours, and higher precipitation at the end of the preceding year negatively affect the last stages of the horse chestnut leaf miner’s life cycle. This results in fewer pests overwintering in the pupa stage and a weaker infestation in the following year [11,12]. In the most natural locations of white horse chestnuts in humid mountain valleys, a low degree of tree colonization by the horse chestnut leaf miner was observed [13]. It can be assumed that the high level of air humidity is the reason why the pest occurs in fewer numbers in these places than on specimens growing in large cities, where the average air humidity is lower by approx. 10% compared to suburban areas and the average temperature is higher by 6–7 °C [14].

**Figure 1:**
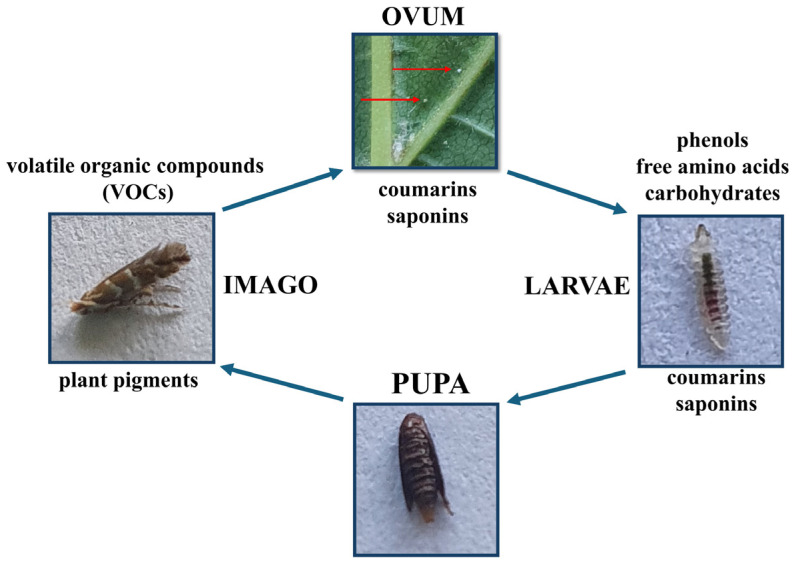
The figure shows the stages of the development of a horse chestnut leaf miner and the chemical compounds that may affect the pest in various stages of development, modifying the size of the moth population. Blue arrows indicate successive developmental stages, while red arrows represent *C. ohridella* eggs. (photo by M. Paterska).

A plant’s resistance or susceptibility to pests results from the interaction between the host plant and the pests. The essential element in this relationship is the chemical composition of the host plant leaves, which is pivotal in facilitating or hampering feeding [15,16,17]. Several leaf chemical compounds can affect *Cameraria ohridela* in various stages of their life cycle, as shown in Figure 1. Imagines can recognize both visual and volatile stimuli [18,19,20,21,22].

The larvae of this leaf-mining insect cannot change their feeding location; they have to feed on leaves selected by the ovipositing females [2]. For this type of feeding, good-quality food that is abundant in carbohydrates and amino acids is essential. On the other hand, secondary metabolites such as saponins, coumarins, and phenolic compounds are toxic to various life stages [23].

In previous studies, our research team demonstrated that the chemical composition of chestnut leaf blades affects the susceptibility of trees to the horse chestnut leaf miner *Cameraria ohridella* [24]. Trees with leaves rich in carbohydrates exhibit greater susceptibility to *C. ohridella*. These findings were confirmed by Bogoutdinova et al. [25], who additionally observed in the same experiment that trees resistant to *C. ohridella* had leaves with a higher tannin content. A significant role in the defensive response of horse chestnuts against the horse chestnut leaf miner (*Cameraria ohridella*) is also attributed to catechins [26]. A crucial role in plant–insect interactions is played by volatile organic compounds (VOCs), which are regarded as signals mediating the communication between plants and other organisms. These substances may act as attractants, which can allow host selection by insects, or as repellents discouraging feeding [27,28,29]. Affecting the behavior of insects, VOCs may determine plants’ resistance or susceptibility to herbivores [30,31,32,33,34].

It has been demonstrated that the volatile compounds present on leaves influence the feeding preferences of butterflies as well as the egg-laying process [35,36]. VOCs are emitted by plants constitutively, and their synthesis is triggered as a result of pest feeding [37,38]. VOCs usually occur as a complex mixture of low-molecular-weight lipophilic compounds originating from different biosynthetic pathways. According to their biosynthetic origin and chemical structure, VOCs can be grouped into isoprenoids or terpenoids [39,40,41]. A specific group of VOCs is green leaf volatiles (GLVs), which are derivatives of C18 fatty acids formed in a particular lipoxygenase (LOX) pathway. The level of GLVs in intact plant tissues is low, but they are rapidly synthesized when plant tissues are damaged by a variety of stress factors, including insect herbivores [42]. GLVs play a crucial role in the interactions between plants and Lepidoptera pests by attracting them to host plants and stimulating the continued feeding on leaf tissues [43,44].

The interaction between chestnut trees and *Cameraria ohridella* starts from identifying and locating host plants by imago, thanks to VOCs emitted by chestnut trees. Females of *Cameraria ohridella* can recognize 20 VOCs emitted by *Aesculus* trees. VOCs directly affect females’ behavior and influence the quantity and distribution of the eggs deposited on the leaves [18,19,20]. The profile of VOCs emitted by chestnut leaves changes during feeding. Gradually, VOCs appear, acting as a deterrent to female moths (furanoid and decanal). More valuable to females are green, undamaged leaves that produce VOCs acting as attractants and stimulating them to oviposit, because larvae hatched from eggs deposited on healthy, undamaged leaves have an abundance of food [18,22].

The main objective of this study was to examine the differences in the profile of leaf VOCs emitted by *Aesculus* trees, which differ in susceptibility to *C. ohridella.* The susceptibility of the examined trees to the pest was assessed based on the degree of leaf coverage with mines. The study was conducted over three growing seasons and focused on single mature tree specimens, which are the representatives of *Aesculus* species considered susceptible and resistant to *C. ohridella.* These *Aesculus* trees grow in the same area and thus in similar environmental conditions. The differences in resistance to *C. ohridella* between these *Aesculus* trees have been confirmed during our several-year observations. Such an approach design has limitations in terms of generalizing the results. However, it excluded other potential sources of variation, such as the intra-species diversity of the host or environmental conditions.

Keeping in mind that the moth can go through up to five generations during the growing season [45], the assessments were carried out across 11–12 dates from the end of April to the end of September. The impact of pest feeding (the appearance of the larvae and first mines) on the changes in the profile of leaf VOCs was also evaluated. An exploratory analysis was used to compare the profile of leaf VOCs with those resistant to pests. The essence of the research was the observation of nature. That is why we conducted several years of research on selected *Aesculus* trees growing in natural conditions.

## 2. Results

### 2.1. Weather Conditions

The average air temperature for the entire growing season (from April to September) was the lowest in 2015 and the highest in 2014 (Table 1). At the beginning of the growing season (April and May), the highest monthly average air temperature was in 2014, and the lowest was in 2015. A gradual increase in monthly average air temperature was observed from April to July in 2014 and 2016 but from April to August in 2016. The months with the highest average temperatures were July 2014 and 2016 and August 2015. In all years, the decrease in monthly average air temperature was observed in September.

The average humidity level for the entire growing season (from April to September) was the highest (91.18%) in 2016 and the lowest (71.13%) in 2015 (Table 2). Both in 2014 and 2016, there was an increase in humidity levels from April to September. The increase was higher in 2014 (from 46.15% to 100%) but lower in 2016 (from 79.7% to 90.4%). However, in 2015, no clear downward or upward trend in humidity was observed. The average speed of wind for the entire growing season was the highest in 2016 and the lowest in 2015 (Table 2). In all years of research, wind speed has been higher in April and lower in September. However, throughout the growing season, a variable trend of changes in wind speed was found.

### 2.2. Damage of Leaves

**Figure 2:**
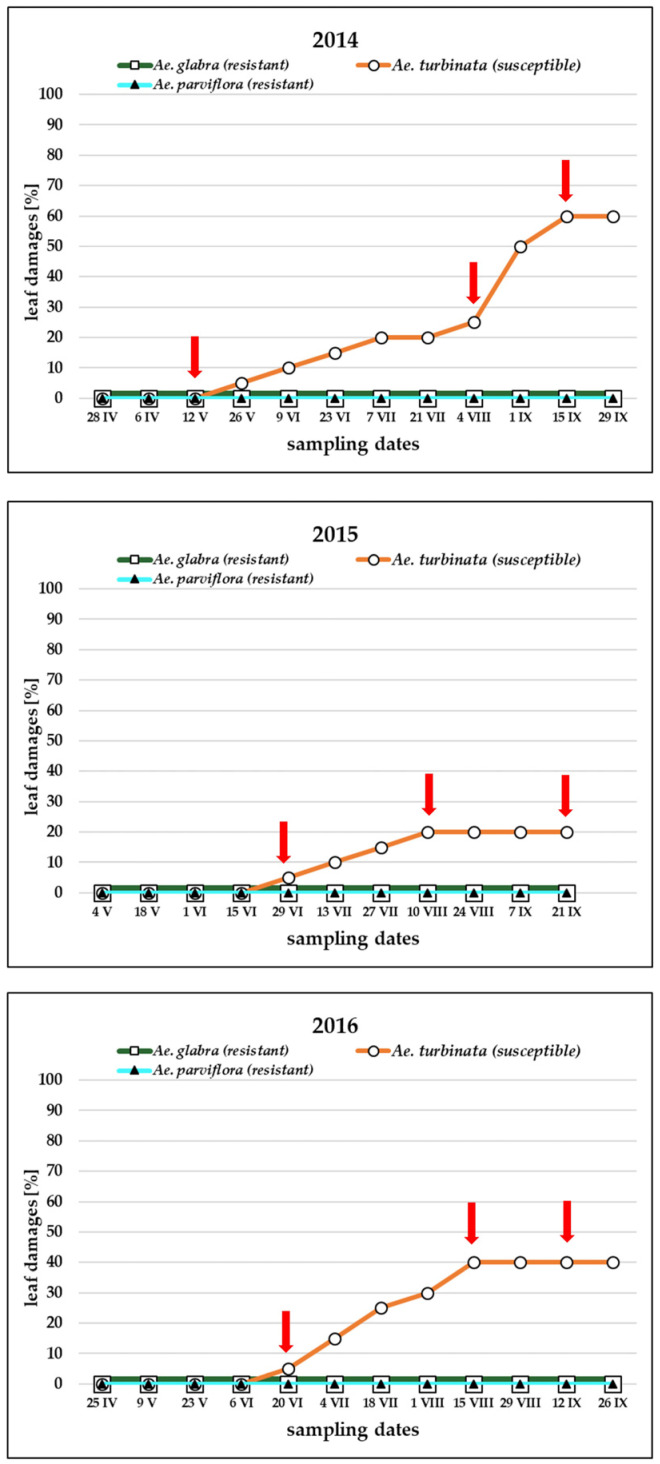
The damage of leaf blades in Japanese horse chestnut (*Aesculus turbinata*), *Ohio buckeye* (*Ae. glabra*), and Bottlebrush buckeye (*Ae. parviflora*) in the years 2014, 2015, and 2016. The Arabic numerals shown in the graphs represent the day of the month, while the Roman numerals indicate the month. For example, “28 IV” corresponds to 28 April. The red arrows indicate the starting dates of subsequent generations of the pest.

The leaf blades of the pest-resistant tree of *Ae. glabra* and the tree of *Ae. parviflora* were not inhabited by female pest, and the feeding symptoms in the form of leaf damage were not observed (Figure 2). In the pest-susceptible tree *Ae*. *turbinata*, a gradual increase in leaf cover with mines was found during the growing season. The date of appearance of the first larvae and mines and the degree of leaf damage varied from year to year.

In all years, the leaves of *A. turbinata* were inhabited by three generations of the pest. In 2014, the larvae of the subsequent generations appeared on 12 V, 4 VIII, and 15 IX. In 2015, they appeared on 29 VI, 10 VIII, and 21 IX, but in 2016, on 20 VI, 15 VIII, and 12 IX. The Arabic numerals shown in the graphs represent the day of the month, while the Roman numerals indicate the month. For example, “28 IV” corresponds to 28 April. The first mines in 2014 were observed on 12 May, and at the end of September, leaf blade damages were at the level of 60%. In the following years, the first mines did not appear until June 29 and June 20 in 2015 and 2016, respectively. In these years, only 20% and 40% of damage to leaf blades was detected at the end of September, respectively.

The Spearman rang correlation coefficient revealed a medium but statistically significant positive correlation (r = 0.447, *p* = 0.007) between air temperature and degree of *Ae. turbinata* leaf damage. It shows that the temperature significantly affected the intensity of feeding, which resulted in damage to the leaves.

### 2.3. Comparative Analysis of the Profile of VOCs in Leaves

The quantity of the VOCs detected in the leaf blades of the analyzed *Aesculus* trees varied from one growing season to another (Table 3). More volatile compounds were identified in 2014 and 2015 than in 2016.

Some differences were also observed between pest-resistant trees (*Ae. glabra*, *Ae. parviflora)* and the pest-susceptible tree (*Ae. turbinata)*. Especially in 2014, a more significant number of VOCs were found in *Ae. turbinata* than in *Ae. glabra* and *Ae. parviflora*. It is worth noting that the pest-susceptible tree of *Ae. turbinata* was characterized by the highest number of VOCs in 2014 when leaf damage was also the greatest (Figure 2). GC-MS analysis also showed that some VOCs occurred on all dates of the growing season and others occurred only on one or more dates.

### 2.4. Andrews Curve Analysis

**Figure 3:**
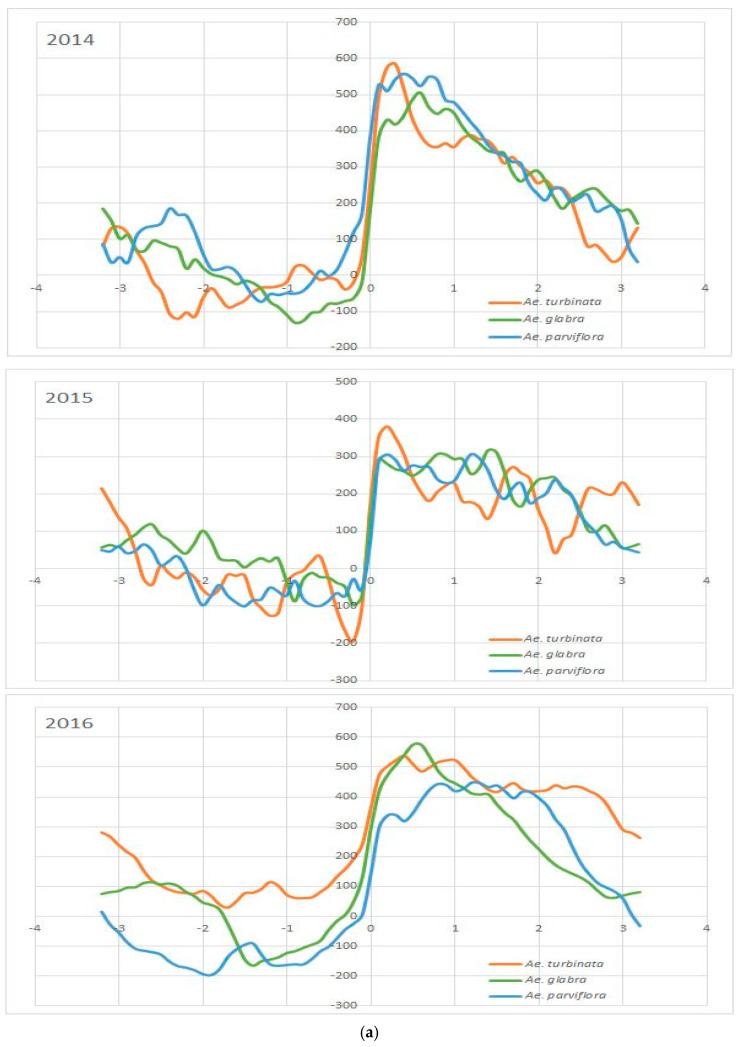

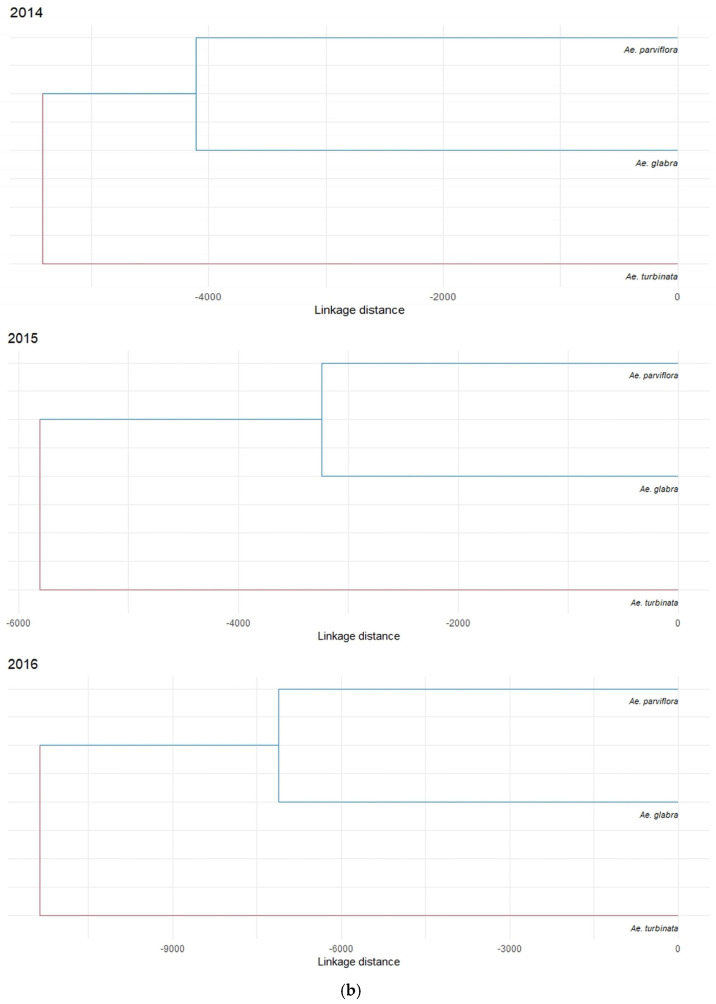
(**a**). Andrews curves created for *Ae. glabra*, *Ae. parviflora*, and *Ae. turbinata* for all years of study (2014, 2015, and 2016). (**b**). The Hierarchical cluster analysis for Andrews curve data. Red line—the first cluster resistant tree; blue line—susceptible tree.

On the graph, every line represents a volatile profile of one *Aesculus* tree. The shape of the Andrews curve characterizes the qualitative and quantitative profile of leaf VOCs in all growing seasons for one *Aesculus* individual (Figure 3). The more similar the profile of VOCs is, the more similar the Andrews curves are. The shape of the lines characterizing resistant trees (*Ae. parviflora* and *Ae. glabra*) and susceptible ones (*Ae. turbinata*) are different. However, the lines that characterize two resistant trees, i.e., *Ae. glabra* and *Ae. parviflora*, are similar. Furthermore, the cluster analysis conducted for the coordinates of points obtained using the Andrews method showed that the resistant trees and the susceptible tree are located in separate groups (Figure 3b). These relationships are repeated every year, although the shape of the curves characterizing the VOCs profile of the leaves of each tree is different in subsequent years. The obtained results revealed the differences in the profiles of leaf VOCs between susceptible trees of *Ae. turbinate* and two resistant trees (*Ae. glabra*, *Ae. parviflora*) in all examined growing seasons.

### 2.5. Analysis of the Quantitative and Qualitative Profile of VOCs Using the DCA Method

**Figure 4:**
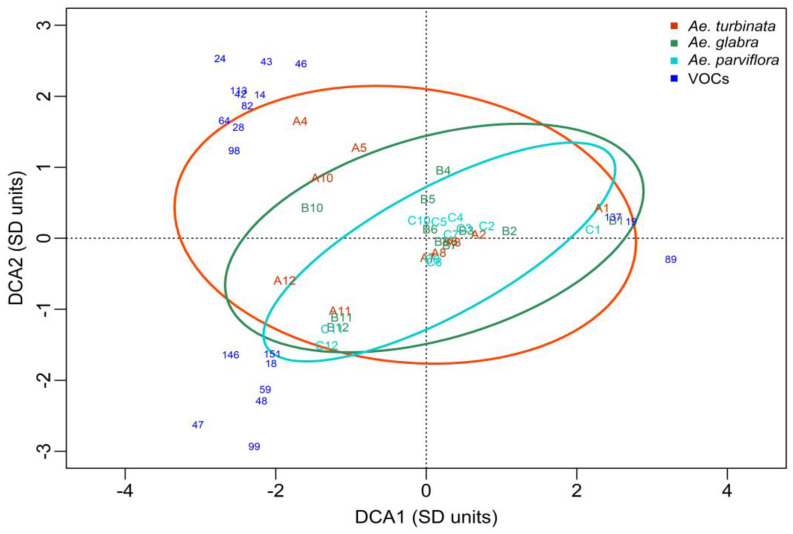
DCA plot, including the profile of the VOCs in examined trees in 2014. A—*Ae. turbinata* orange line and letters; B—*Ae. glabra* green line and letters; C—*Ae. parviflora* light blue line and letters. Numbers (1–12) added to letters A, B, and C represent the sampling dates. Blue numbers—VOCs that most strongly distinguish trees and terms whose distance from the center of the coordinate system was >2.5. The numbers 24, 46, 82, 98, and 99 indicate groups of VOCs with the same coordinates.

The DCA plot presenting the analysis of the results obtained in 2014 shows an ordering of the points concerning dates rather than trees (Figure 4). In all chestnut trees, there is a large distance between the first sampling date (28 IV; A1, B1, C1) and the last two dates (15 IX; A11, B11, C11, and 29 IX; A12, B12, C12). A large distance is observed, especially in the susceptible *Ae. turbinata* (A) tree, along the DCA1 axis between the first and twelfth dates, and also along the DCA2 axis between the fourth and eleventh dates. This indicates that the qualitative and quantitative VOC profiles of *A. turbinata* leaves are different on these dates (a few common VOCs are present). The differences in the profile of leaf volatile compounds between the first (1) and the last date (12) in the resistant trees *Ae. glabra* (B) and *Ae. parviflora* (C) are slightly smaller. The positions of the points representing the VOCs profile show that there are no differences between susceptible and resistant trees for most of the dates (1–3, 6, 7, 11) (the Andrews curves were quite similar but the cluster analysis showed that the resistant and susceptible trees were in another cluster).

However, it is worth mentioning that the points representing dates 4 and 5 for the susceptible tree (A) are significantly distant from the points representing these dates for the resistant trees (B, C). Some differences can also be seen between the susceptible (A) and resistant trees (B, C) on date 12. On the above dates, differences in the VOC profile between the susceptible tree, *Ae. turbinate*, and the resistant trees, *Ae. glabra* and *Ae. parviflora*, are evident. These differences correspond to some extent with the feeding dates of the pest. The start of feeding was observed on 12 May, which corresponds to the third sampling date. The dates for the start of feeding of subsequent generations were 4 VIII (9th sampling date) and 15 IX corresponds to the 11th sampling date.

The ellipse defines the 95% confidence area of the tree *Ae. turbinata* (A) and it covers the largest area and contains most of the areas of the ellipses determined for the resistant trees *Ae. glabra* (B) and *Ae. parviflora* (C). This means that the qualitative and quantitative profile of the leaf VOCs of susceptible trees was different from that of resistant trees. The confidence ellipses determined for the pest-resistant trees are not identical, indicating some differences in the profile of VOCs between these trees. These differences, however, are not as great as those between the susceptible *Ae. turbinata* and the resistant *Ae. glabra* and *Ae. parviflora*. A two-factor multivariate permutation analysis of variance using the Adonis method showed that the differences between the tested trees in their volatile compound profiles are not statistically significant (*p* = 0.075; F = 1.67; df = 2; 20). In contrast, the differences between the terms are statistically significant (*p* = 0.0010; F = 5.30; df = 10; 20). But in the cluster analysis, the differences between trees were visible.

The DCA (Figure 4), in addition to the differences between trees on some dates, identified a group of compounds that affected the leaf VOC profiles of the studied trees in 2014. The graph shows only the compounds that contributed the most to the separation between trees and dates (distance from the center of the coordinate system greater than 2.5 units). Some of these compounds had the same coordinates and overlapped. The number of one compound from such a group is marked on the plot. All compounds, along with the name and number, as well as the date of occurrence and the tree on which it was found, are included in Table 4.

A high level of Phytol (137) was found in all trees on the first date. This was also found in all trees but at a much lower levels on dates 2 and 3, as well as in resistant trees on dates 4 and 5 (Figure 4, Table 4). In the susceptible tree (*Ae. turbinata*), a group of VOCs (24, 54, 79, 86, 100, 114, 159) was identified on date 4 (after the first generation of the pest began feeding), which were present only in this tree and only on this date (A4). All of these compounds in the plot overlap (have the same coordinates) and have been marked with a common number, 24; their names are given in Table 4. Similarly, on date 5, a group of VOCs were identified that occurred only in the susceptible tree (46, 87, 120, 133, 155). These compounds have the same coordinates and are marked by the common number 46 in the plot; their names are given in Table 4. Only in the susceptible tree, and only on dates 4 and 5, a compound named 26-Nor-5-cholesten-3, beta,-ol-25-one (43) was identified, and on dates 4 and 10, two compounds, i.e., 2,6,10,14,18-Pentamethyl-2,6,10,14,18-eicosapentaene (42) and Methyl tetradecanoate (113) were found. In addition, 9-octadecenoic acid (64) was found to be highest on dates 4 and 5 in the susceptible tree. This compound was present in this tree on dates 10 and 12, as well as on the 10th date in the resistant tree. In contrast, 11,13-Dimethyl-12-tetradecen-1-ol acetate (14), which was present in the leaves of the susceptible tree on date 3 and 4, was also found in the leaves of the resistant tree (C) on date 11. No VOCs were identified that were specific to the resistant trees alone (*Ae. glabra and Ae. parviflora*) and were not present in the susceptible tree. Only Phytol (137), which was present in all trees in the first three terms, was exclusively present on dates 4 and 5 in both resistant trees and on date 7 in the resistant tree *Ae. glabra.* These results indicate that the susceptible tree, especially on the sampling dates (4, 5) when the feeding of the first generation of the pest was observed, had a higher number of characteristic VOCs than the resistant trees. In addition, two VOCs (19 and 89) were present only at the beginning of the growing season (date 1) in all trees. However, a large group of compounds (18, 28, 47, 48, 59, 82, 98, 99, 116, 141, 143, 146, 147, 151) were present in all trees at the last sampling dates (11, 12). Their names were not given because they did not affect the distinction of the studied trees.

In summary, the VOCs identified in the DCA as having the most impact on tree differentiation are those exclusively present in the susceptible tree at sampling dates close to the feeding dates of the subsequent pest generations. These are the compounds numbered 14, 24, 42, 43, 46, 54, 64, 79, 86, 87, 100, 113, 114, 120, 133, 155, and 159. Two of them (14 and 64) were also present in the resistant *Ae. parviflora* and *Ae. glabra* trees, respectively, on the most recent sampling dates. The compound with a higher proportion in the leaf profile of resistant trees than in that of the susceptible tree was Phytol (137).

**Figure 5:**
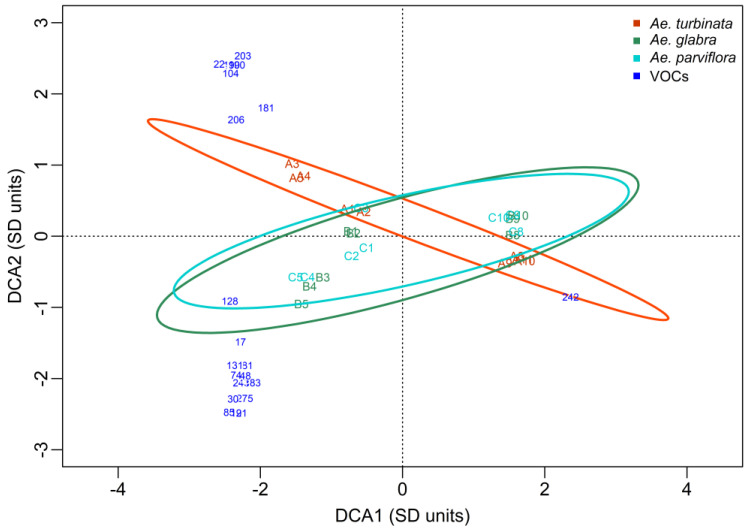
DCA plot including the profile of the VOCs in examined trees in 2015. A—*Ae. turbinata* orange line and letters; B—*Ae. glabra* green line and letters; C—*Ae. parviflora* light blue line and letters. Numbers (1–11) added to letters A, B, and C represent the sampling dates. Blue numbers—VOCs that most strongly distinguish trees and terms whose distance from the center of the coordinate system was > 2.5. The numbers 22, 81, 85, 104, and 203 indicate groups of VOCs with the same coordinates.

In the next growing season (2015), the difference between sampling dates, as was seen in the DCA plot, was not as clear as in 2014 (Figure 5). The major differences (distance between points) were recorded along the DCA1 axis in all trees between terms 4 and 5 and final terms 8, 9, 10, and 11. The distance along the DCA2 axis of the points representing resistant and susceptible trees on dates 3, 4, and 5 also indicates the differences in the VOC profile between these trees on the above dates. Some differences in VOC profiles are also noticeable between resistant trees and susceptible ones at the beginning of the growing season (sampling dates 1 and 2). In 2015, feeding started on 29 VI and corresponded to sampling date 5. On this date, the VOC profile was similar to that on dates 3 and 4 (Figure 5) but differed from dates 1 and 2. The other feeding dates were 1 VIII (9th sampling date) and 21 IX (11th sampling date). Thus, as in 2014, differences in the VOC profile between the resistant trees and the susceptible one occurred on dates close to the pests’ feeding dates.

The ellipse defines the 95% confidence area of trees not damaged by the pest. The similarity between *Ae. glabra* and *Ae. parviflora* suggests that the VOC profiles of these trees are also similar. This profile was not the same as that of the leaves of the susceptible tree *Ae. turbinata*. The differences between trees and dates shown in the DCA plot were confirmed statistically using a two-factor multivariate permutation analysis of variance. Statistically significant differences were found both between trees (*p* = 0.0220, F = 3.04, df = 2;14) and dates (*p* = 0.0010, F = 6.88, df = 8;14). These results are compatible with the results given in the cluster analysis made using Andrew curves.

The DCA plot (Figure 5) shows the group of VOCs with the most affected profiles in the studied trees in 2015. Only the compounds with the strongest differentiation are shown, which indicate extreme trees and terms (distance from the center of the system greater than 2.5 units). The quantity of VOCs that influenced the profile of trees in 2015 was considerably higher than in 2014. As in 2014, some of these compounds had the same coordinates and overlapped. The numbers and names of the noticeable VOCs, as well as the dates and trees in which they occurred, are included in Table 4. In the susceptible tree, a large group of VOCs were identified on dates 3 through 5 that were not present in resistant trees. Important compounds for distinguishing this tree include the compound numbered 190, present from dates 3 to 5. As well as being present only on the third date, three compounds (22, 148, 253) with the same coordinates are marked in the DCA graph with the number 22 (Figure 5). Important for distinguishing this tree were also five compounds (203, 221,274, 216, 226) present on date four and three compounds (104, 20, 286) present on date five. These compounds on the DCA plot were groups with the same coordinates and were marked with the common numbers 203 and 104, respectively. In addition, compounds valuable for their profile were identified in resistant trees. These included two compounds (48, 243) present in both of these trees on dates 3 through 5. It is also present on dates 4 and 5, only in the *Ae. parviflora* tree compound number 30, as well as a group of five compounds (81, 149, 192, 217, 231) with the same coordinates and three compounds (85, 202, 237) with the same coordinates. On the DCA plot, these groups are numbered 81 and 85, respectively. Three compounds (121, 191, 275) present only in the resistant tree *Ae. glabra* on dates four and/or five were also identified (Table 5).

**Figure 6:**
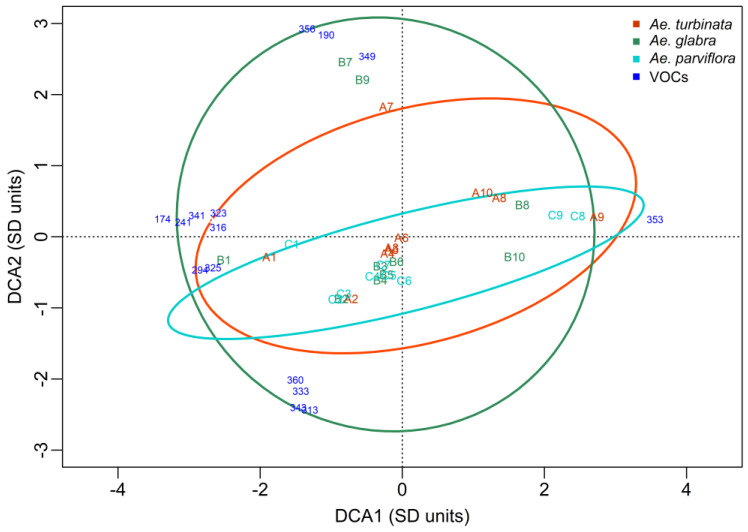
DCA plot including the profile of the VOCs in examined trees in 2016. A—*Ae. turbinata* orange line and letters; B—*Ae. glabra* green line and letters; C—*Ae. parviflora* light blue line and letters. Numbers (1–11) added to letters A, B, and C represent the sampling dates. Blue numbers—VOCs that most strongly distinguish trees and terms whose distance from the center of the coordinate system was > 2.5. The numbers 174, 313, and 316 indicate groups of VOCs with the same coordinates.

In 2016, the diversity along the DCA1 and DCA2 axes was bigger than in 2014 and 2015 (Table 6). The largest distance occurs along the DCA1 axis between the first sampling date (A1) and the ninth sampling date (A9) for the susceptible tree *Ae. turbinata* (Figure 6). This indicates valuable differences in the quantitative and qualitative profile of VOCs exist between these dates (similar to 2014). Some minor differences are seen between the first and last terms in the resistant trees. The largest distance is found along the DCA1 axis between points representing the 1st, 8th, and 10th sampling dates in the *Ae. glabra* resistant tree (B). In contrast, in the resistant tree *Ae. parviflora* (C), this was shown between the 1st, 8th, and 9th sampling dates. In addition, in this tree, some differences in the VOC profile occurred between the 2nd date and the 7th as well as 9th dates, indicated by the large distance between points representing these dates along the DCA2 axis. The points of the susceptible tree (*Ae. turbinata*) corresponding to dates 3–6 are very close to the center of the coordinate system. This means that the VOC profile of this tree on the above dates is similar. Very close to this group are the points corresponding to these dates in resistant trees (*Ae. parviflora*, *Ae. glabra*). On dates 3, 4, 5, and 6, points designating the VOC profile of the studied trees are close to each other, which indicates a slight variation in this profile on the mentioned dates exists. More important differences between trees were noted on later dates (7–10). In 2016, the start of feeding was dated 20.VI and corresponds to the fifth sampling date. Subsequent dates for the start of feeding by subsequent generations are 15 VIII and 12 IX, corresponding to the 9th and 11th sampling dates.

In 2016, the confidence ellipses determined for resistant trees differed significantly. The ellipse defines the 95% confidence area of the resistant tree *Ae. glabra*, which covers the largest area and occupies most of the areas determined for the other trees. In contrast, the ellipse for the susceptible tree *Ae. turbinata* occupies most of the area of the ellipse of the resistant tree *Ae. parviflora*. Based on the results of the two-factor multivariate permutation analysis of variance, significant differences were shown both between terms (*p* = 0.0010; F = 5.59; df = 9;17) and between trees (*p* = 0.0080; F = 3.06; df = 2;17), and the cluster analysis confirmed that.

The DCA identified a group of VOCs that strongly influenced the formation of their profile in the studied trees. All distinctive VOCs were present on the initial or last sampling dates (Table 6). These were the three compounds (323, 341, 362) present only in both resistant trees on the first date and one compound (360) present on the second date. Moreover, two compounds (343 and 356) were found on dates 1 and 2 only in resistant *Ae. glabra*. On the first date and only in this tree, there was a group of three compounds (174, 321, 532) with identical vectors in the DCA plot (Figure 6), labeled with the number 174. In the susceptible tree (A), it was possible to distinguish between two groups of characteristic VOCs with identical vectors marked in the DCA plot with the numbers 313 and 316 (Figure 6). These were the three compounds (316, 337, 342) present on the first date and the two compounds (313, 317) present on the second date. The other VOCs listed in Table 6 were present in all trees on similar dates.

## 3. Discussion

One of the factors influencing the intensity of the horse chestnut leaf miner’s infestation is weather conditions, in particular, air temperature, sunlight, and air humidity [11,12]. This pest is a xerophilous insect, so low air temperatures, especially at the turn of April and May when the moths fly out, as well as high air humidity, can reduce its population [46,47]. Insolation and temperature affect the rate of the development of individual stages of the horse chestnut leaf miner’s life cycle.

In the present study, in the first year of the investigation (2014), the average air temperatures for each month of the growing season (except for June) were higher compared to the average temperatures for each month in the following two seasons (2015 and 2016). In 2014, the highest degree of leaf blade cover with mines was observed in the susceptible *Ae. turbinata* tree, which indicates a more intensive pest infestation than in other seasons. It is worth noting that the higher air temperature in May 2014, compared to other years for this month, may have affected the earlier start of pest infestation, which resulted in more leaf damage. The lowest average air temperatures in individual months and lower average air temperature than in other years were in May 2015. In this growing season, the lowest degree of leaf blades covered with mines was also recorded, which indicates that the pests’ feeding intensity was lower than in other years. The observations presented above confirm the influence of atmospheric conditions, and especially temperature, on the degree of leaf colonization by the horse chestnut leaf miner in the susceptible horse chestnut tree *Ae. turbinata*. In the examined resistant horse chestnut trees, *Ae. parviflora* and *Ae. glabra*, no leaf damage resulting from pest feeding was observed.

Atmospheric conditions can also affect the chemical composition of plants, including a plant’s defensive compounds, called secondary metabolites [48,49].

In the growing season of 2014, during which the highest average air temperatures were recorded for individual months, the highest number of VOCs was detected in each of the examined trees compared to the following years. However, in 2015, when the lowest average air temperature was recorded, more volatile compounds were detected than in the slightly warmer year 2016. This suggests that the richness of VOC profiles may be dependent on temperature. Still, this is not the only factor influencing the number of volatile compounds emitted by chestnut leaves during the growing season. Another factor could be the degree of pest feeding. In our previous studies, we found that horse chestnut leaf miner feeding increased the content of anthocyanins and phenolic compounds in both white horse chestnut and Japanese horse chestnut leaves [24]. Hanaka [50] showed a higher content of phenolic compounds in white horse chestnut leaves with symptoms of feeding than in leaves without these symptoms. The results presented in this paper indicate that the degree of pest feeding may also be a factor affecting changes in the VOC content of leaves. The highest number of VOCs in susceptible Japanese horse chestnuts (*Ae. turbinata*) was found in 2014, when the greatest leaf damage occurred, representing the highest intensity of pest feeding. As a result, it is not possible to conclusively determine the relative contribution of each factor to the increased emission of volatile organic compounds (VOCs). In this growing season, during which the most damage was recorded, the amount of VOCs in Japanese horse chestnut tree was also higher than the resistant *Ae. glabra* and *Ae. parviflora* trees. Unfortunately, this relationship was not observed in the other years, when the number of VOCs in the leaves of susceptible chestnut trees was similar or only slightly lower than that in the leaves of resistant chestnut trees.

The data analysis through the Andrews curve method allowed for a preliminary comparison of the VOC profiles of the leaves of two trees resistant to the pest (*Ae. glabra* and *Ae. parviflora*) and a susceptible tree (*Ae. turbinata*). The VOC profiles of the studied trees changed every year, and few of the identified VOCs were detected in all three years of the experiment. However, in all years of the study, the VOC profiles of the leaves of the tree damaged by the pest (*Ae. turbinata*) differed from the VOC profiles of the two resistant trees (*Ae. glabra* and *Ae. parviflora*). These observations indicate that the factors that affected the VOC profiles of the examined trees could be both the atmospheric conditions in a given growing season and species characteristics. VOCs are emitted by all plant taxa, both constitutively and in response to biotic and abiotic stress [51]. They are regarded as signals (or cues) mediating communication between plants and other organisms, as well as “bioactive” compounds that elicit responses in the receiver. VOCs play a crucial role in plant–biotic interactions, such as host selection by insects [27], acting as feeding, habitat, and oviposition cues for both beneficial insects and herbivores [52,53,54]. The profile of VOCs varies among plant species, cultivars, varieties, genotypes, tissues within the same plant [55,56,57], and in response to biotic and abiotic factors, e.g., herbivory or drought [58,59]. These properties make VOCs a good source of information about the identity and status of the emitting plant to other organisms.

The use of the DCA allowed for a more detailed comparison between the VOC profiles of the tested trees on successive dates of the growing season and the determination of the effect of feeding on the change in the volatile compound profile of the leaves of the susceptible tree. The results show significant differences in the quantitative and qualitative profile of leaf VOCs at the beginning of the growing season, before the infestation of the susceptible tree with *Ae. turbinata*, compared to the results from the end of the growing season, when the leaf blades were damaged to the greatest extent. The VOCs that most significantly differentiated the profile of the examined trees are those found only in the susceptible tree. These compounds were present on sampling dates close to the feeding periods of subsequent pest generations. This was particularly evident in 2014, when the leaf blades were damaged to the greatest extent. In the susceptible tree of *Ae. turbinata*, 15 volatile compounds were identified and detected on the feeding dates of the first generation and up to a month after the start of feeding. It is possible that these compounds appeared as a result of damage to cell membranes, as a result of tissue biting, and then attracted subsequent individuals of *C. ohridella*, leading to intensive colonization and, consequently, important damage to the leaves of *Ae. turbinata*. These compounds were not detected in any of the resistant chestnut trees (*Ae. glabra*, *Ae. parviflora*). In 2015, twelve VOCs were detected in susceptible trees which were not present in resistant trees. As in 2014, these compounds were present on dates immediately before the onset of feeding, at the onset of feeding, or up to 2 weeks after the larvae began to bite tissues. In the following year (2016), feeding of the pest was less intense and the least VOCs were detected—there were about half as many as in the previous years of the experiment. The DCA identified five volatile compounds present in the leaf profile of the susceptible tree. They were detected only on the dates preceding the start of pest feeding.

As a result of the conducted studies, differences in the volatile organic compound profiles between the susceptible tree (*Ae. turbinata*) and the two resistant trees (*Ae. glabra* and *Ae. parviflora*) were observed, which can be explained within the context of ecological theories. According to the plant defense theory, plants develop and refine defensive mechanisms, including the production of volatile compounds, as part of their chemical defense, in order to reduce pressure from herbivores [60]. In the case of the susceptible tree *Ae. turbinata*, a higher diversity of VOCs in 2014 could represent a response to the intensified attacks by the pest. However, these defense mechanisms proved ineffective in limiting the feeding activity of *C. ohridella* larvae. On the other hand, the resistant trees *Ae. glabra* and *Ae. parviflora* emitted volatile compounds that could act as deterrents to female moths searching for a host plant. It should be noted that the larvae of the horse chestnut leaf miner are unable to change feeding sites, as their legs have atrophied, so the choice of host plant by the females determines the access to high-quality food for the larvae [61,62].

An alternative plant defense cost–benefit theory suggests that the production of defensive VOCs is associated with energetic costs for plants, which must balance the allocation of metabolic energy between defense against pathogens and herbivores or growth and reproduction [61]. In 2014, when higher average temperatures were observed, the trees may have altered their VOC profiles in response to the increasing pressure from the pest. Such adaptive changes could also be related to the rising costs of VOC production under more extreme conditions, such as increased temperature [61].

Differences in VOC production between susceptible and resistant trees may also result from the plant–pest interaction theory, which proposes that plants can “communicate” with herbivores by producing chemical compounds that influence insect feeding preferences [62]. Thus, the variability in VOC profiles in response to the intensity of leaf miner feeding may represent a form of ecological communication between the plant and the pest, where more resistant trees produce VOCs that may repel pests or limit their ability to feed [61].

The variable VOC profiles across years may also indicate plant adaptations to changing environmental conditions, as previous studies have suggested that plants adjust their defense mechanisms in response to changing climatic conditions [61].

The horse chestnut leaf miner causes significant damage annually by damaging leaf blades or completely defoliating horse chestnut trees. The first signs of damage appear in early May, while the complete destruction of the leaf blades often occurs by August. The reduction in the photosynthetically active leaf area leads to a decrease in photosynthetic intensity [63]. It has been estimated that energy losses caused by inhibited photosynthesis due to the reduced photosynthetically active leaf area amount to 37% during a single growing season. This contributes to reduced shoot growth, smaller fruit size, lower seed quality, or complete lack of fruiting. Damage caused by *C. ohridella* significantly disfigures trees which are intended to serve an esthetic role in urban and rural landscapes [64]. To date, the proposed methods for protecting horse chestnut trees from *C. ohridella* have proven ineffective. The use of synthetic plant protection products conflicts with the global trend toward reducing chemical inputs in horticulture and agriculture. Biological methods have been insufficient in effectively controlling pest populations, while sticky traps are non-selective and can capture pollinators, which is highly undesirable. Moreover, the thorough removal of leaves containing overwintering pupae is impractical to implement [22,65].

Considering the annually expanding range of *C. ohridella* and the lack of effective control measures, it is crucial to understand the biochemical interactions between the host plant and the pest. This knowledge is essential for developing effective and environmentally safe methods to reduce pest populations.

## 4. Materials and Methods

### 4.1. Plant Material

The trees of the genus *Aesculus* used in this study grow in the Botanical Garden of the Adam Mickiewicz University in Poznan (52°25′ N, 16°53′ E) in semi-shaded places. These include an individual of *Ae*. *turbinata* susceptible to the pest and one individual of *Ae*. *glabra* and *Ae. parviflora* resistant to the pest (Table 7). None of the studied trees was treated against diseases and pests, either before or during the research. The experimental objects were individual chestnut (*Aesculus*) trees, and the experimental units (biological replications) were leaves harvested from different branches of a given tree.

### 4.2. Sample Collection

Fully developed compound leaves were randomly chosen every 14 days, at the same time of day (between 8 and 9 a.m.), from April to September 2014, 2015, and 2016. Only green leaf parts, without mines, were taken for analysis. The collected material was put into glass jars and transferred to the laboratory within no more than 1 h.

### 4.3. Weather Data

The measurements were performed by the meteorological Weather Station installed at the Experimental Station HD2003, located at 31 Starołęcka Street in Poznan. Results are presented as the means for each month in the period from March to October. Additionally, the minimum and maximum temperature values for each month are given.

### 4.4. Leaf Damage Assessment

The degree of leaf damage was evaluated visually by the same person according to the method described by Wojciechowski and Baranowski [66]. At each sampling date, 15 fully developed compound leaves were collected from each examined chestnut tree. For each compound leaf, the percentage of the leaf blade area covered with mines, indicative of the feeding activity of the horse chestnut leaf miner (*Cameraria ohridella*), was visually assessed individually. Subsequently, the arithmetic mean of the measurements was calculated separately for each tree. All observations were consistently performed by the same researcher.

The juvenile larvae formed round light green mines, while the larvae, before pupation, fed in dark brown elongated corridors caused by the drying of the epidermis. The differences in the shape, size, and coloration of the mines and larvae made it clear as to which generation a larva feeding in a particular mine belonged. Every time, 15 compound leaves from each tree were evaluated. After measurement, the leaves were used for chemical analyses.

### 4.5. Determination of Leaf Volatile Organic Compounds (VOCs)

The profile of VOCs in the leaf extracts was determined by the modified gas chromatography-mass spectrometry (GC-MS) method [67]. Leaf samples (500 mg) were placed in a centrifuge tube, and 5 cm^3^ of HPLC-grade methanol was added. All was mixed, and homogenate was filtered to clear extracts. The filtrate was stored in a sealed glass vial at 4 °C for further GC-MS analysis. The evaluation of the profile of leaf VOCs was carried out through an Agilent 7890 GC system coupled with an Agilent 5975 MS system (Santa Clara, CA, USA). The GC was equipped with a silica capillary column, Supelco SPB-1 30 m × 0.25 id (0.25 µm) (Sigma-Aldrich St. Louis, MO, USA). Helium (99.9999%) was used as carrier gas at a flow rate of 1.0 mL/min. Then, 1 μL of the sample was injected in split mode. The detector and injector temperatures were maintained at 300°C. The oven temperature was programmed to 50 °C for 1 min, then increased at 15 °C/min to 230 °C, and then at 25 °C/min to 320 °C. The mass scan range (*m*/*z*) was 33–400 amu.

The identification of the VOCs appearing in the profile was carried out based on the National Institute of Standards and Technology (NIST) Mass Spectral Library. The credibility of all identifying compounds was higher than 75%. The concentration of each VOC was expressed as a percentage of the peak area relative to the total peak area from the GC-MS analysis of the sample. All determinations were carried out in three biological replications. Each biological replicate consisted of a methanol extract prepared from individual leaf samples.

### 4.6. Statistical and Explorative Analyses of the Results

#### Spearman Rank Correlation Coefficient

The effect of air temperature on leaf damage caused by *C. ohridella* feeding in the *Ae. turbinata* tree susceptible to the pest was tested using the Spearman rang correlation coefficient (r). The calculation was made using the R package version 4.4.2.

### 4.7. Analysis of the Results Using the Andrews Curve Method

A graphical representation method for multidimensional data was employed to characterize the differences in the quantitative and qualitative profiles of leaf VOCs among the examined trees. This approach transforms multiple dimensions (compounds) into individual curves within a coordinate system, as described in [36]. In this representation, the horizontal axis corresponds to the arguments of the functions defining the Andrews curves, spanning from −π to π, while the vertical axis indicates the values of these functions. The resulting curves provide a visual depiction of the VOC profiles, incorporating both qualitative and quantitative chromatographic data. These curves encapsulate information about the percentage contribution of each detected VOC to the total pool across all sampling dates. A separate curve was generated for each analyzed tree (object), ensuring that the same sequence of traits (compounds) was maintained throughout. This method enables a clear assessment of the differences between the examined objects [37]. The curves were created using a custom program written in Scilab 6.0, an open source software platform used for numerical computation. For the coordinates obtained from this analysis, the hierarchical clustering (with Manhattan distances and Ward method) analysis was used to see how trees are grouped.

### 4.8. Detrended Correspondence Analysis (DCA)

DCA, as described by Hill [68], is a frequently used statistical method in ecological studies. Research carried out in this area, which involves determining the abundance of a given species (plant, animal) in selected locations, gives the observation matrices, the elements of which are the number of individuals of a given species present at a given site (location). Because individuals of a given species do not occur in every site, many zeros appear in the data matrix. Such a matrix is called a sparse matrix. Traditional multidimensional analysis (for example, correspondence analysis) for this type of data leads to the so-called horseshoe effect. DCA eliminates this inconvenience hindering data interpretation. The table of our observations includes all identified VOCs in the three studied chestnut trees at the subsequent determination dates. The elements of this table are the percentage content of a given compound in the entire profile. As not all compounds were present in all trees and on all dates, many zeros appeared in the table.

Thus, our results of the profile of the leaf VOCs of the examined chestnut trees give a sparse data matrix, which suggests the use of the DCA method for data analyses. The results of the DCA are the biplot representing trees (A, B, C) at different sampling dates (1 to 11 or 12), with the points and leaf VOCs as the numbers. The axes on a DCA plot are measured in units of standard deviation, SD, where one SD unit represents the average distance over which species reach their maximum occurrence and then begin to decline. If there are uncorrelated points on opposite sides of the axis, then they have very few VOCs in common, and the longer the axis, the fewer the VOCs they have in common. If the axis is longer than 3, trees on opposite sides of the axis can be expected to have no VOCs in common, but an axis of a length of 1 shows that points on opposite sides of the axis have at least 50% different VOCs [69]. The results of the DCA reveal that the length of the DCA1 axis is longer than 3 in all years. Meanwhile, the length of the DCA2 axis was shorter than 3 only in 2015, but it was longer than 1 (Table 8).

The position of VOC on the DCA graph (blue numbers in Figure 4, Figure 5 and Figure 6) shows which trees have the highest content of this compound on a given date. Points representing trees and dates lying on the side opposite to the axis of the identified VOC indicate that the tree contains little or none of this compound. The further away a given VOC is from the center of the coordinate system, the greater the impact on distinguishing between trees and dates is. The trees furthest from the beginning of the coordinate system have a higher abundance of a given VOC. Only VOCs whose distance from the center of the coordinate system is higher than 2.5 units have been included in the DCA diagrams. In addition, confidence ellipses have been plotted in which all observations for a given tree are included with 95% confidence.

### 4.9. Two-Factor Multivariate Permutation Analysis of Variance (Adonis)

The DCA is presented in a plot from which the statistical significance of differences between groups (*Aesculus* trees, sampling dates) is impossible to obtain. To verify whether the examined trees differ significantly in VOC profile, a two-factor multivariate permutation analysis of variance (Adonis) was used. If the two-factor multivariate permutation analysis of variance showed significant differences, a multilevel pattern analysis was performed. This made it possible to analyze the relationship between the profile of VOCs and the homogeneous groups formed from the analyzed objects (trees) and dates.

Both statistical and explorative analyses were conducted in the R environment using procedures from the vegan 2.6-8 package [70] and the ‘interspecies’ package version 1.7.15 [71].

## 5. Conclusions

In conclusion, the present study has shown the differences in the profile of VOCs between susceptible and resistant *Aesculus* trees. However, this profile varied from year to year. No compounds were found that were present annually in the profiles of the tested trees. A key finding was that the VOC profile of the susceptible tree changed in response to pest feeding.

## Figures and Tables

**Table 1:** Air temperature (°C) during growing season.

Month	Air Temperature [°C]								
	2014			2015			2016		
	Averages	Min.	Max	Averages	Min.	Max	Averages	Min.	Max
April	11.93	−0.50	23.30	7.45	−0.80	25.10	8.75	−0.60	24.70
May	15.55	6.20	30.00	11.95	3.10	25.80	14.65	5.30	29.20
June	14.50	7.70	33.10	16.40	8.70	32.80	18.80	8.70	33.90
July	22.94	11.30	35.30	15.50	10.00	35.80	18.90	10.80	32.80
August	18.99	8.50	33.80	18.80	8.10	38.50	17.90	9.80	32.7
September	16.55	2.70	27.20	14.95	4.90	35.30	15.30	5.50	31.70
Average	16.74	5.98	30.45	14.18	5.67	32.22	15.72	6.59	30.83

**Table 2:** Humidity level and speed of wind during the growing season.

Month	Average Humidity [%]			Average Wind Speed [m∙s^−1^]		
	2014	2015	2016	2014	2015	2016
April	46.15	80.15	79.70	0.59	0.86	0.95
May	64.75	65.10	86.65	0.84	1.06	0.71
June	84.80	59.85	100.00	0.95	0.63	0.90
July	91.20	72.55	100.00	0.27	0.59	0.97
August	91.45	69.85	90.40	0.14	0.43	0.35
September	100.00	79.25	90.35	0.30	0.32	0.29
Average	79.73	71.13	91.18	0.51	0.65	0.69

**Table 3:** The number of VOCs detected in green leaves of the examined *Aesculus* trees.

Genotype	The Number of All Identified VOCs		
	2014	2015	2016
	Resistant		
*Ae. glabra*	59	54	39
*Ae. parviflora*	64	65	46
	Susceptible		
*Ae. turbinata*	77	53	34

**Table 4:** VOCs that most strongly affected by the distinction between trees and dates in 2014 (VOCs with the same coordinates are combined in one table cell). A—*Ae. turbinata*, B—*Ae. glabra*, C—*Ae. parviflora.*

No. of VOC	Name of VOC	Trees	Dates
14	11,13-Dimethyl-12-tetradecen-1-ol acetate	A C	3–4 11
24	15-Tetracosenoic acid		
54	8,11-Octadecadienoic acid,		
79	cis-11-Hexadecenal		
86	Cyclopentadecanone, 2-hydroxy-	A	4
100	E-15-Heptadecenal		
114	Methyl 7-methylhexadecanoate		
159	Trimethyl [4-(1-methyl-1-methoxyethyl)phenoxy]silane		
42	2,6,10,14,18-Pentamethyl-2,6,10,14, 18-eicosapentaene	A	4, 10
43	26-Nor-5-cholesten-3,beta,-ol-25-one	A	4–5
46 87 120 133 155	3-Eicosene, (E)- Cyclopentane, (4-octyldodecyl)- Octadecanamide Oxiraneundecanoic acid, 3-pentyl-, Tricosanoic acid	A	5
64	9-Octadecenoic acid,	A B	4–5, 10, 12 10
113	Methyl tetradecanoate	A	4, 10
137	Phytol	A B C	1–3 1–5, 7 1–5
146	Squalene	A B	3, 12 10

**Table 5:** VOCs that most strongly affected by the distinction between trees and dates in 2015 (VOCs with the same coordinates are combined in one table cell). A—*Ae. turbinata*, B—*Ae. glabra*, C—*Ae. parviflora.*

No. of VOC	Name of VOC	Trees	Dates
17	13-Borabicyclo [7.3.0]tridecane, -propoxy-, (Z)- or (E)-	A B C	4–5 4 4–5
22 148 253	1,4-Eicosadiene Tetracosanoic acid, methyl ester Ethanedioic acid, dimethyl ester	A	3
30	2-Hexadecene, 3,7,11,15-tetramethyl-, [R-[R*,R*-(E)]]-	C	4–5
48	3,7,11,15-Tetramethyl-2-hexadecen- 1-ol	B C	3 5
74	Bicyclo [4.1.0]heptane, 3-methyl-	B C	3 4–5
81 149 192 217 231	Cyclododecyne Tetradecanal 11-Tricosene 5(1H)-Azulenone, 2,4,6,7,8,8a-hexahydro-3,8-dimethyl-4-(1-methyl ethylidene)-, (8S-cis)- Benzenepropanenitrile, 3,4-dimethoxy-	C	4
85 202 237	Cyclopentadecane 2(1H)-Naphthalenone, octahydro-4a- methyl-7-(1-methylethyl)-, (4a.alpha.,7.beta.,8a.beta.)- Bicyclo [10.1.0]tridec-1-ene	C	5
104 204 286	Furo [3,4-c]pyridine-3,4(1H,5H)-dione, 6-methyl- 2,6-Difluorobenzoic acid, 2-bromo-4-fluorophenyl ester R-(-)-1,2-propanediol	A	5
121	Octadecanal	B	5
128	Oleyl Alcohol	AC	5
131	Oxime-, methoxy-phenyl-_	AB	5
181	1,2-Benzenediol	A B C	1–5 1–5 3–5
183	1,2-Cyclohexanedione	B	4
190	11,14,17-Eicosatrienoic acid, methyl ester	A	3–5
191	11-Hexadecen-1-ol, acetate, (Z)- 2,4a,8,8-	B	5
203 216 221 226 274	Tetramethyldecahydrocyclopropa[d]naphthalene 4-Hydroxy-3-methylacetophenone 7-[2,6-Dichlorobenzyl]-4-chloropyrrolo [2,3-d]pyrimidine 9-Octadecenal, (Z)- N-Methyl-7-azabicyclo(2,2,1)hept-2-ene	A	4
206	2,6-Difluorobenzoic acid, 4-nitrophenyl ester	A C	4 5
242	Cholesta-4,6-dien-3-ol, (3.beta.)-	A	10–11
243	cis,cis,cis-7,10,13-Hexadecatriena	B C	3–5
275	Nonanoic acid, 9-(3-hexenylidenecyclopropylidene)-, 2-hydroxy-1-(hydroxymethyl)ethyl ester, (Z, Z, Z)-	B	4–5

**Table 6:** VOCs that most strongly affected the distinction between trees and terms in 2016 (VOCs with the same coordinates are combined in one table cell). A—*Ae. turbinata*, B—*Ae. glabra*, C—*Ae. parviflora.*

No. of VOC	VOC	Trees	Dates
174 321 352	alpha,-Methyl mannofuranoside 6,9,12-Octadecatrienoic acid, methyl ester Naphthalene, decahydro-2,6-dimethyl-3-octyl-	B	1
190	11,14,17-Eicosatrienoic acid, methyl ester	A B	7 7, 9
233	Benzofuran, 2,3-dihydro-	A, B, C	1
241	Catechol	A, B	1
294	[1,1’-Bicyclopropyl]-2-octanoic acid, 2’-hexyl-, methyl	A B C	1 1–2 1–2
298	12-Hydroxy-3-keto-bisnor-4-cholenic acid	A, B	2
300	1-Heptatriacotanol	A, B, C	1
307	2,6,10,14-Hexadecatetraen-1-ol, 3,7,11,15-tetramethyl-,	A, B	2
313 317	2-Tridecyne 3-Trifluoroacetoxypentadecane	A	2
338	Cyclopropanecarboxylic acid, 2,2-dimethyl-3-(2-propenyl)-,	AB	1
316 337 342	3-Methylmannoside Cyclopropanebutanoic acid, 2-[[2-[[2-[(2-pentylcyclopropyl) Dodecanoic acid, 2,3-bis(acetyloxy)propyl ester	A	1
323	7-Hexadecenal, (Z)-	BC	1
325	8,11,14-Eicosatrienoic acid, (Z, Z, Z)-	AB C	1–2 1
333	Cholest-4-ene-3,6-dione	A, B C	2 1–2
335	Cinnamic acid, 4-hydroxy-3-methoxy-, (5-hydroxy-2-hydroxymethyl-	ABC	2
341	Cyclopropanetetradecanoic acid, 2-octyl-, methyl	B, C	1
343	Dodecanoic acid, 3-hydroxy-	B	2
349	Heptacosanoic acid, methyl ester	A, C	7
353	Palmitaldehyde, diisopentyl acetal	A C	9 8
356	trans-Farnesol	B	7
360	Undec-10-ynoic acid, dodecyl ester	B, C	2
362	Z,Z-8,10-Hexadecadien-1-ol	B, C	1

**Table 7:** *Aesculus* trees used in the study.

Genotype	Susceptibility/Resistance	Location
*Ae*. *glabra*	Resistant	Botanical Garden UAM, plot G13
*Ae. parviflora*	Resistant	Botanical Garden UAM, plot G12
*Ae*. *turbinata*	Susceptible	Botanical Garden UAM, plot G18

**Table 8:** The length of the DCA1 and DCA2 axes in subsequent years based on detrended correspondence analysis.

Year	2014		2015		2016	
DCA axes	DCA1	DCA2	DCA1	DCA2	DCA1	DCA2
Axis lengths	4.4067	3.1607	3.2717	1.9722	5.2486	3.3359

## Data Availability

The raw data supporting the conclusions of this article will be made available by the authors on request.

## References

[1] Csóka G. (2003). Levélaknák és Levélaknázók/Leaf Mines and Leaf Miners.

[2] Maria B. (1988). Mining Insects of Poland: A Key for Identification Based on Leaf Mines. [Owady Minujące Polski. Klucz do Oznaczania na Podstawie Min.].

[3] Kenis M., Girardoz S., Avtzis N., Kamata N. Finding the area of origin of the horse-chestnut leaf miner: A Challenge. Proceedings of the IUFRO Kanazawa 2003 Forest Insect Population Dynamics and Host Influences.

[4] Sefrova H., Skuhravy Z. (2001). Dispersal of the horse-chestnut leafminer *Cameraria ohridella* Deschka & Dimić 1986, in Europe: Its course, ways and causes (Lepidoptera: Gracillariidae). Entomol. Z..

[5] Didmanidze E.A., Supatashvili A., Goginashvili N. (2000). Butterflies of Georgian Forests. Cameraria ohridella Continues to Spread in Europe.

[6] Gninenko Y.I., Muhamadiev N.S., Ashikbaev N.Z. (2017). *Cameraria ohridella*: The first record in Central Asia. Russ. J. Biol. Invasions.

[7] Gubin A.I. (2021). Four invasive alien phytophagous insects new to Armenia. Phytoparasitica.

[8] Gilbert M., Guichard S., Freise J., Grégoire J.C., Heitland W., Straw N., Tilbury C., Augustin S. (2005). Forecasting *Cameraria ohridella* invasion dynamics in recently invaded countries: From validation to prediction. J. Appl. Ecol..

[9] Girardoz S., Quicke D.L.J., Kenis M. (2007). Factors favouring the development and maintenance of outbreaks in an invasive leaf miner *Cameraria ohridella* (Lepidoptera: Gracillariidae): A life table study. Agric. For. Entomol..

[10] Bystrowski C., Celmer-Warda K., Tarwacki G. (2008). Effects of horse chestnut (*Aesculus hippocastanum* L.) site on horse chestnut leafminer (*Cameraria ohridella* Deschka & Dimić) parazytoids appearance and number in Central Poland. For. Res. Pap..

[11] Cedro A., Nowak G. (2022). Influence of horse-chestnut leaf miner invasion on the growth-climate relationship of common horse-chestnut trees from north-western Poland. Sylwan.

[12] Bogoutdinova L.R., Tkacheva E.V., Konovalova L.N., Tkachenko O.B., Olekhnovich L.S., Gulevich A.A., Baranova E.N., Shelepova O.V. (2023). Effect of Sun Exposure of the Horse Chestnut (*Aesculus hippocastanum* L.) on the Occurrence and Number of *Cameraria ohridella* (Lepidoptera: Gracillariidae). Forests.

[13] Walas Ł., Dering M., Ganatsas P., Pietras M., Pers-Kamczyc E., Iszkuło G. (2018). The present status and potential distribution of relict populations of *Aesculus hippocastanum* L. in Greece and the diverse infestation by *Cameraria ohridella* Deschka & Dimić. Plant Biosyst.—Int. J. Deal. Asp. Plant Biol..

[14] Łukasiewicz S., Oleksyn J. (2007). Heterogeneity of Spatial Meteorological Traits and Their Effects on horsechestnut (Aesculus hippocastanum L.) Development in Urban Conditions of Poznan.

[15] Curir P., Galeotti F., Dolci M., Barile E., Lanzotti V. (2007). Pavietin, a coumarin from *Aesculus pavia* with antifungal activity. J. Nat. Prod..

[16] Ferracini C., Curir P., Dolci M., Lanzotti V., Alma A. (2010). Aesculus pavia foliar saponins: Defensive role against the leafminer *Cameraria ohridella*. Pest Manag. Sci..

[17] Lanzotti V., Termolino P., Dolci M., Curir P. (2012). Paviosides A–H, eight new oleane type saponins from *Aesculus pavia* with cytotoxic activity. Bioorg. Med. Chem..

[18] Johne A.B., Weissbecker B., Schütz S. (2006). Volatile emissions from *Aesculus hippocastanum* induced by mining of larval stages of *Cameraria ohridella* influence oviposition by conspecific females. J. Chem. Ecol..

[19] Johne A.B., Weißbecker B., Schütz S. (2006). Microorganisms on *Aesculus hippocastanum*—Olfactory perspective of *Cameraria ohridella* (Deschka & Dimic). Mitteilungen Dtsch. Ges. Für Allg. Angew. Entomol..

[20] Johne A.B., Sprauer S., Weißbecker B., Schütz S. (2006). Influence of flower odour compounds on oviposition of the horse chestnut leaf miner *Cameraria ohridella* (Deschka & Dimic). Mitteilungen Dtsch. Ges. Für Allg. Angew. Entomol..

[21] Tomczyk A., Ptak A., Titova M., Oreshnikov A., Nosov A. (2007). Effect of *Polyscias filicifolia* bailey extracts on the behavior of the leaf Miner *Cameraria ohridella* deschka and dimic on horse chestnut trees. Acta Biol. Cracoviensia Ser. Bot..

[22] Svatoš A., Kalinová B., Hrdý I. (2009). *Cameraria ohridella*: 10 years of sex pheromone and kairomone research. J. Appl. Entomol..

[23] Harborne J.B. (1996). Plant Secondary Metabolism. Plant Ecology.

[24] Paterska M., Bandurska H., Wysłouch J., Molińska-Glura M., Moliński K. (2017). Chemical composition of horse-chestnut (Aesculus) leaves and their susceptibility to chestnut leaf miner *Cameraria ohridella* Deschka & Dimić. Acta Physiol. Plant..

[25] Bogoutdinova L.R., Shelepova O.V., Konovalova L.N., Tkachenko O.B., Gulevich A.A., Baranova E.N., Mitrofanova I.V. (2024). Susceptibility of Different Aesculus Species to the Horse Chestnut Leaf Miner Moth: Chemical Composition and Morphological Features of Leaves. J. Zool. Bot. Gard..

[26] Materska M., Pabich M., Sachadyn-Król M., Konarska A., Weryszko-Chmielewska E., Chilczuk B., Staszowska-Karkut M., Jackowska I., Dmitruk M. (2022). The Secondary Metabolites Profile in Horse Chestnut Leaves Infested with Horse-Chestnut Leaf Miner. Molecules.

[27] Bruce T.J.A., Wadhams L.J., Woodcock C.M. (2005). Insect host location: A volatile situation. Trends Plant Sci..

[28] Dicke M. (2009). Behavioural and community ecology of plants that cry for help. Plant Cell Environ..

[29] Szakiel A. (2015). Plant Volatile Compounds—Structure, Biosynthesis, and Role in Environmental Interactions. Kosmos.

[30] Ruther J., Mayer C.J. (2005). Response of garden chafer, *Phyllopertha horticola*, to plant volatiles: From screening to application. Entomol. Exp. Appl..

[31] Fernandez P.C., Meiners T., Björkman C., Hilker M. (2007). Electrophysiological responses of the blue willow leaf beetle, *Phratora vulgatissima*, to volatiles of different *Salix viminalis* genotypes. Entomol. Exp. Appl..

[32] Büchel K., Malskies S., Mayer M., Fenning T.M., Gershenzon J., Hilker M., Meiners T. (2011). How plants give early herbivore alert: Volatile terpenoids attract parasitoids to egg-infested elms. Basic Appl. Ecol..

[33] Engelberth J., Contreras C.F., Dalvi C., Li T., Engelberth M. (2013). Early transcriptome analyses of Z-3-Hexenol-treated zea mays revealed distinct transcriptional networks and anti-herbivore defense potential of green leaf volatiles. PLoS ONE.

[34] Scala A., Allmann S., Mirabella R., Haring M.A., Schuurink R.C. (2013). Green Leaf Volatiles: A Plant’s Multifunctional Weapon against Herbivores and Pathogens. Int. J. Mol. Sci..

[35] Mofikoya A.O., Yli-Pirilä P., Kivimäenpää M., Blande J.D., Virtanen A., Holopainen J.K. (2020). Deposition of α-pinene oxidation products on plant surfaces affects plant VOC emission and herbivore feeding and oviposition. Environ. Pollut..

[36] Wang C., Wang D., Zeng F., Chen L., Zhao X., Zhu X., Yao J., Li Y. (2024). Identification on Key Volatiles Contributed to Oviposition Preference of *Plodia interpunctella* (Hübner, 1813) (Lepidoptera: Pyralidae) from High and Normal Oleic Varieties of Peanut. Insects.

[37] Das A., Lee S.-H., Hyun T.K., Kim S.-W., Kim J.-Y. (2013). Plant volatiles as method of communication. Plant Biotechnol. Rep..

[38] Effah E., Holopainen J.K., Clavijo McCormick A. (2019). Potential roles of volatile organic compounds in plant competition. Perspect. Plant Ecol. Evol. Syst..

[39] Brilli F., Hörtnagl L., Bamberger I., Schnitzhofer R., Ruuskanen T.M., Hansel A., Loreto F., Wohlfahrt G. (2012). Qualitative and quantitative characterization of volatile organic compound emissions from cut grass. Environ. Sci. Technol..

[40] Yang Z., Baldermann S., Watanabe N. (2013). Recent studies of the volatile compounds in tea. Food Res. Int..

[41] Vivaldo G., Masi E., Taiti C., Caldarelli G., Mancuso S. (2017). The network of plants volatile organic compounds. Sci. Rep..

[42] Matsui K., Engelberth J. (2022). Green Leaf Volatiles—The Forefront of Plant Responses Against Biotic Attack. Plant Cell Physiol..

[43] He J., Halitschke R., Schuman M.C., Baldwin I.T. (2021). Light dominates the diurnal emissions of herbivore-induced volatiles in wild tobacco. BMC Plant Biol..

[44] Joo Y., Schuman M.C., Goldberg J.K., Wissgott A., Kim S.-G., Baldwin I.T. (2019). Herbivory elicits changes in green leaf volatile production via jasmonate signaling and the circadian clock. Plant Cell Environ..

[45] D’costa L., Koricheva J., Straw N., Simmonds M.S.J. (2013). Oviposition patterns and larval damage by the invasive horse-chestnut leaf miner *Cameraria ohridella* on different species of Aesculus. Ecol. Entomol..

[46] Wilczyński S., Podlaski R. (2007). The effect of climate on radial growth of horse chestnut (*Aesculus hippocastanum* L.) in the Świętokrzyski National Park in central Poland. J. For. Res..

[47] Shvydenko I.M., Stankevych S.V., Zabrodina I.V., Bulat A.G., Pozniakova S.I., Goroshko V.V., Hordiiashchenko A.Y., Matsyura A.V. (2021). Diversity and distribution of leaf mining insects in deciduous tree plantations. A review. Ukr. J. Ecol..

[48] Holopainen J.K., Virjamo V., Ghimire R.P., Blande J.D., Julkunen-Tiitto R., Kivimäenpää M. (2018). Climate Change Effects on Secondary Compounds of Forest Trees in the Northern Hemisphere. Front. Plant Sci..

[49] Yang Y., Battesti M.-J., Costa J., Dupuy N., Paolini J. (2018). Volatile components as chemical markers of the botanical origin of Corsican honeys. Flavour Fragr. J..

[50] Hanaka A., Dresler S., Mułenko W., Wójciak M., Sowa I., Sawic M., Stanisławek K., Strzemski M. (2023). Phenolic-Based Discrimination between Non-Symptomatic and Symptomatic Leaves of Aesculus hippocastanum Infested by *Cameraria ohridella* and *Erysiphe flexuosa*. Int. J. Mol. Sci..

[51] Holopainen J. (2004). Multiple functions of inducible plant volatiles. Trends Plant Sci..

[52] Pare P.W., Tumlinson J.H. (1999). Plant volatiles as a defense against insect herbivores. Plant Physiol..

[53] Pichersky E., Gershenzon J. (2002). The formation and function of plant volatiles: Perfumes for pollinator attraction and defense. Curr. Opin. Plant Biol..

[54] Dudareva N., Negre F., Nagegowda D.A., Orlova I. (2006). Plant Volatiles: Recent Advances and Future Perspectives. Crit. Rev. Plant Sci..

[55] Krips O.E., Willems P.E., Gols R., Posthumus M.A., Gort G., Dicke M. (2001). Comparison of cultivars of ornamental crop Gerbera jamesonii on production of spider mite-induced volatiles, and their attractiveness to the predator *Phytoseiulus persimilis*. J. Chem. Ecol..

[56] Jönsson M., Lindkvist A., Anderson P. (2005). Behavioural responses in three ichneumonid pollen beetle parasitoids to volatiles emitted from different phenological stages of oilseed rape. Entomol. Exp. Appl..

[57] Kappers I., Aharoni A., Herpen T., Luckerhoff L., Dicke M., Bouwmeester H. (2005). Genetic Engineering of Terpenoid Metabolism Attracts Bodyguards to Arabidopsis. Science.

[58] Holopainen J.K., Gershenzon J. (2010). Multiple stress factors and the emission of plant VOCs. Trends Plant Sci..

[59] Clavijo McCormick A., Unsicker S.B., Gershenzon J. (2012). The specificity of herbivore-induced plant volatiles in attracting herbivore enemies. Trends Plant Sci..

[60] Turlings T.C., Tumlinson J.H. (1992). Systemic release of chemical signals by herbivore-injured corn. Proc. Natl. Acad. Sci. USA.

[61] Heil M., Karban R. (2010). Explaining evolution of plant communication by airborne signals. Trends Ecol. Evol..

[62] Fürstenberg-Hägg J., Zagrobelny M., Bak S. (2013). Plant Defense against Insect Herbivores. Int. J. Mol. Sci..

[63] Myśkow E., Sokołowska K., Słupianek A., Gryc V. (2021). Description of Intra-Annual Changes in Cambial Activity and Differentiation of Secondary Conductive Tissues of Aesculus hippocastanum Trees Affected by the Leaf Miner *Cameraria ohridella*. Forests.

[64] Holoborodko K.K., Seliutina O.V., Ivanko I.A., Alexeyeva A.A., Shulman M.V., Pakhomov O.Y. (2021). Effect of *Cameraria ohridella* feeding on Aesculus hippocastanum photosynthesis. Regul. Mech. Biosyst..

[65] Lesovoy N., Fedorenko V., Vigera S., Chumak P., Kliuchevych M., Strygun O., Stoliar S., Retman M., Vagaliuk L. (2020). Biological, Trophological, Ecological and Control Features of Horse-Chestnut Leaf Miner (*Cameraria ohridella* Deschka & Dimic). Ukr. J. Ecol..

[66] Wojciechowski T., Baranowski T. (2011). Effect of soil conditioners on soil water availability dynamics and health status of horse chestnut. Prog. Plant Prot..

[67] Sermakkani M., Thangapandian V. (2012). Gc-Ms Analysis of Cassia Italica Leaf Methanol Extract. Asian J. Pharm. Clin. Res..

[68] Hill M.O., Gauch H.G. (1980). Detrended Correspondence Analysis: An Improved Ordination Technique. Vegetatio.

[69] Gauch H.G. (1973). A Quantitative Evaluation of the Bray-Curtis Ordination. Ecology.

[70] Oksanen J., O’Hara B., Kindt R., Stevens H. (2010). Vegan: Community Ecology Package.

[71] Cáceres M.D., Legendre P. (2009). Associations between species and groups of sites: Indices and statistical inference. Ecology.

